# Linking policies and regulations to sustainable finance for the promotion of urban agriculture: Evidence from micro and small businesses

**DOI:** 10.1016/j.heliyon.2024.e31938

**Published:** 2024-05-24

**Authors:** Goshu Desalegn, Anita Tangl, Maria Fekete-Farkas, Girma Gudisa, Anita Boros

**Affiliations:** a Doctoral School of Economics and Regional Sciences, Hungarian University of Agriculture and Life Sciences, 2100, Godollo, Hungary; b Department of Accounting and Finance, Kotebe University of Education, Addis Ababa, P.O. Box 16417, Ethiopia; c John von Neumann University, Doctoral School of Management and Business Administration, 6000, Kecskemét, Izsáki str. 10, Hungary; d Institute of Agricultural and Food Economics, Hungarian University of Agriculture and Life Sciences, Szent István, 2100, Godollo, Hungary; e Department of Accounting and Finance, Rift Valley University, Addis Ababa, Ethiopia

**Keywords:** Urban agriculture, Sustainable finance, Government policies and regulations

## Abstract

Urban agriculture is closely tied to several of the Sustainable Development Goals. It can play a critical role in helping to achieve these goals by promoting sustainable food production and consumption, reducing greenhouse gas emissions, and creating more sustainable cities. It is also considered a pathway for overcoming food security in urban areas. However, this needs to be integrated with policies and regulations supported by sustainable finance. Due to COVID-19, conflict, and lack of infrastructure in Ethiopia, several challenges must be addressed to promote urban agriculture to aid food security. Hence, this study is conducted to examine how government policies and regulations promote urban agriculture through sustainable finance in Ethiopia. The study employed both an explanatory research design and a mixed research approach. Multi-stage sampling techniques that include (Simple random sampling techniques and judgmental sampling were used. The data was collected from selected micro and small enterprises engaged in urban agriculture. The structural and measurement model is estimated with the help of smart-pls software version 4. The study's finding implies that government policies and regulations have an insignificant effect on urban agriculture. At the same time, the study finding implies that sustainable finance has an important mediating role between urban agriculture and government policies. Hence, the impact of government policies and regulations on urban agriculture is found to have an indirect effect. Based on the study's findings, the study recommends that all stakeholders promote innovation and entrepreneurship that promote urban agriculture through sustainable finance.

## 1 Introduction

Urban agriculture is gaining popularity on a global scale because both domestic and international studies show that it has the potential to increase urban food security significantly [1]. An increasing population and other crises like Climate change, pandemics, and conflicts in some parts of the world have all threatened the agri-food industry's growth and food security. As a result, they put tremendous pressure on policymakers worldwide to shift their focus on developing a more sustainable and resilient agri-food industry [2]. However, the transition to sustainable agriculture and agri-food businesses will be challenging to fund in most developing nations' financial systems because of a need for more finance.

Global food demand is predicted to increase by 70 % by 2050, necessitating annual expenditures of at least $80 billion across all value chains [3]. Historically, the food sector needs more funding from banks, microfinance organizations, and institutional investors. As a result, agricultural loans and investment holdings are disproportionally small compared to the GDP share of the industry across the globe [4]. Hence, enhancing the level of the private sector and micro and small enterprises to increase their productivity while reducing environmental impact and taking into account climate risks are found necessary [5].

The micro- and small-business sectors are vital to economic growth and an essential strategy for helping countries escape poverty [6]. They are relatively labor-intensive, and job opportunities are created for a relatively cheap capital cost and increased productivity [7]. However, the management of specific agricultural risks, the high transaction costs, the low effective demand for financing, and the lack of experience financial institutions have in managing agricultural loan portfolios are some of the challenges facing the development of MSEs in the agricultural sector [8]. Government policies and regulations play a crucial role in overcoming the challenges and in promoting urban agriculture by creating a favorable environment for the development and growth of urban agricultural initiatives [9].

Many countries have implemented adequate and effective policies and instruments to provide opportunities for mobilizing private capital for the sector. In doing so, implementing agricultural finance strategies and mechanisms to crowd in private capital and deepening resilient agricultural finance markets were some of the others [10]. More specifically, some African countries (Kenya et al.) have implemented policies and programs to support the growth of urban agriculture. These policies include investment in water management infrastructure, promoting conservation agriculture practices, and developing agroforestry systems in urban areas. Furthermore, additional policies like zoning laws that allow for urban agriculture, tax incentives for urban farmers, and funding for research and development of urban agriculture technologies were considered by some countries in promoting urban agriculture [11]. To that end, Sustainable finance is also one of the most essential ways government policies and regulations may support urban agriculture [12]. The term "sustainable finance" refers to the use of financial instruments and products to support and advance investments that are socially and environmentally responsible [13]. It can be done in the context of urban agriculture by developing financial incentives for the construction of green roofs and other types of urban agriculture, as well as by setting up investment funds and other financial support systems for urban farmers and urban agriculture-related businesses. Strengthening sustainable agriculture finance markets will be a solution for agriculture lenders to access long-term finance, digitize and green their services, manage agriculture risks, and grow agriculture asset classes that are environmentally friendly [14].

In Ethiopia, many of the poorest households find it challenging to pay for food, rent, healthcare, and school fees because of the country's rising cost of living and the capital cities. People are migrating more significantly from the countryside to the towns as they look for employment [15]. The country has recently faced several political, social, and economic challenges, including ethnic and political conflict, poverty, and food insecurity. A conflict in the Tigray region and other corners of the country, widespread violence, and displacement highly reduced agriculture productivity. At the same time, much of the infrastructure was damaged, which resulted in transportation problems. As a result, the issue of food security and urban poverty is becoming a concern. Based on this, it is argued that increasing urban agriculture is critical for overcoming this problem. However, urban agriculture is a popular informal economic activity in many cities [16]. Additionally, society and policymakers do not give more attention to this sector, even though urban farming is a significant contributor to the livelihoods of many city residents.

Some studies are conducted from different perspectives to investigate the challenges and prospects of urban agriculture in Ethiopia [17,18]. However, those studies have yet to investigate the role of government policies and regulations and the direct and indirect mediating part of sustainable finance. As a result, this study is motivated to investigate the direct and indirect role of government policies and regulations in promoting urban agriculture. At the same time, the study will investigate the mediating role of sustainable finance between government policies and regulations and urban agriculture. This study is novel in providing profound empirical evidence to the existing literature on government policies and laws promoting urban agriculture by combining it with sustainable finance, evidenced by micro and small enterprises.

Furthermore, the study is also novel in providing evidence investigated through explanatory research design to see how regression results can give a portion of supportive evidence to the existing literature. Hence, the objective of this study is generally to examine the effect of government policies and regulations on promoting urban agriculture through sustainable finance. The study is divided into five sections, each focusing on a different aspect. Section one is dedicated to discussing the study's introduction, providing an overview of its purpose and background. Section two delves into a comprehensive literature summary of the specific study area. Section three elaborates on the materials and methodology employed throughout the research process. In section four, the results obtained from the study are thoroughly examined and analyzed. Lastly, section five presents the conclusions and recommendations derived from the study's specific findings.

## 2 Literature review

### 2.1 Urban agriculture and government policies and regulations

Urban agriculture refers to cultivating, processing, and distributing food in or around urban areas. It can include various activities, such as community gardens, rooftop gardens, and vertical farms. Urban agriculture provides fresh produce to city dwellers and serves as a tool for community development, environmental sustainability, and economic revitalization [19]. Additionally, urban agriculture can help to address food security issues in urban areas by increasing the availability of fresh, healthy food [20].

Due to the growing demand for fresh agricultural goods and the development of urban food security programs, urban agriculture is considered one strategy for increasing revenue and enhancing nutrition in urban areas [21]. It also offers the potential to make cities greener and healthier. In the 21st century, urban agriculture is mainly becoming famous as it is suitable for outdoor and indoor farming; modern technologies enable agricultural activities inside ordinary buildings in addition to traditional outdoor farming and food production in dedicated greenhouses. Furthermore, advanced technologies are becoming applicable for indoor farming in some buildings to make them favorable for urban agriculture; for example, Sky Green is found in Singapore, and the Suwon vertical farm of South Korea is used for urban agriculture in buildings [22]. Despite technological advancement and its potential for sustainable city development, urban agriculture has many challenges from different aspects of the economy, environment, and society.

The challenges of urban agriculture include not only financing and funding but also providing land use, zoning, and balancing competing demands for land, transportation, traffic congestion, affordable housing, environmental impact, social equity, and ensuring development benefits all members of a community, political will, and stakeholder cooperation, managing population growth and overcrowding and adapting to the challenges of climate change and natural disasters [23].

According to a study by Ref. [24], urban agriculture has received relatively little political support from central and local governments due to its informal status. As a result, many urban farmers need more land access and ownership insecurity and need help to invest in improving their land, inputs, and infrastructure. At the same time, the study by Ref. [25] suggests that policymakers strategically identify sustainable urban agriculture principles to enhance urban agriculture. To this end, the study conducted by Ref. [26] shows that a Lack of knowledge and will among urban planners and policymakers has led to negative attitudes toward urban agricultural activities in the urban planning system.

Generally, urban agriculture faces various difficulties, including land competition, a lack of urban policy directives, unfair land use planning, and decisions regarding land tenure, according to a study conducted [27], which supports this claim. Such barriers are said to be a sign of governance problems. However, these issues could be resolved by eliminating unfair land management decision-making processes, conflicting regulations, and perverse incentives and giving tropical urban agriculture and the environment more technical support. Therefore, by fostering a conducive environment for the growth of urban farming initiatives, government policies, and regulations play a crucial role in promoting urban agriculture. As a result, based on the argument, the following hypothesis assumes the existence of a positive relationship between urban agriculture and government policies and regulations.

H1 Government policies and Regulations have a positive and significant effect on Urban agriculture.

### 2.2 Urban agriculture and sustainable finance

Urban agriculture uses resources such as land, which are in high demand for other urban purposes. City dwellers are becoming aware of the economic importance of urban agriculture. Studies are being conducted to understand better how urban agriculture contributes to urban society's livelihoods [28]. Millions of urban dwellers in many African cities are forced to engage in urban farming to supplement their household income or meet their daily food needs [29]. It is also used to increase food imports in "developed" countries. However, starting urban agriculture has different costs, including start-up costs, operational costs, cost of capital, opportunity costs, managing risks, and competing in the market. If projects for urban agriculture are not given enough assistance, they can have problems getting started [30]. As a result, the urban agriculture business can be expensive due to land, equipment, supplies, and other related expenses.

Furthermore, urban agriculture projects must generate enough revenue to cover costs and provide a return on investment. However, this can be challenging to achieve, especially for small-scale projects. Hence, sustainable finance is necessary to promote micro and small enterprises engaged in the urban agricultural business. Regarding government policies and regulations, Sustainable finance is one of the most important ways to promote urban agriculture [31]. Using financial tools and mechanisms to support and promote environmentally and socially responsible investments is called sustainable finance.

A sustainable finance fund for urban agriculture entails the creation of investment funds and other financial support systems for urban farmers and urban agriculture businesses, as well as financial incentives for the growth of green roofs and different types of urban agriculture [32]. Urban agriculture projects funded with green bonds are one instance of sustainable finance. Green projects are financed with the help of green bonds, such as the construction of green roofs, urban gardens, and other types of urban agriculture. Green bonds are a way for municipalities and other organizations to raise money for urban agriculture projects while also advancing sustainable growth and lowering their carbon footprint [33].

Using impact investing to support urban agriculture is another example of sustainable finance. Impact investing is investing in businesses, organizations, and funds that produce measurable social and environmental effects in addition to a financial return. It can include investing in urban agriculture start-ups and small businesses and assisting in developing urban agriculture technologies and infrastructure [34]. One of the main benefits of sustainable finance is that it can provide access to funding for projects that promote sustainable food production and distribution [35]. It can include investments in infrastructure such as greenhouses, irrigation systems, and equipment for processing and packaging local foods. These investments can help improve urban agriculture enterprises' efficiency and productivity, making them more competitive and sustainable in the long term.

Sustainable finance can also support the development of green spaces and community gardens in urban areas. These green spaces can provide access to land and resources for small-scale farmers and entrepreneurs, helping to promote the development of local food systems. Developing green spaces in urban areas can also help improve water and air quality and encourage biodiversity, providing multiple benefits for the local community. Sustainable financing also aids micro and small businesses enhance their social and environmental performance. Access to technical support, instruction in sustainable agricultural practices, and environmental impact assessment and management tools can help achieve this [36].

Furthermore, innovative business models that advance sustainable food systems can also be encouraged by sustainable finance. Sustainable financing, for instance, can be used to support urban food cooperatives and community-supported agriculture by funding infrastructure and equipment purchases and technical support and training initiatives [37]. These models can support the development of local food systems, expand access to wholesome, and open up new market opportunities for urban agriculture businesses. Including environmental, social, and governance (ESG) considerations in the decision-making process is another crucial component of sustainable finance for urban agriculture. It can ensure that investments in urban agriculture align with long-term sustainability objectives and help develop more dependable, equitable, and sustainable food systems. Sustainable finance can also contribute to the achievement of the United Nations Sustainable Development Goals (SDGs) through the promotion of sustainable food systems, the reduction of poverty and hunger, the improvement of health and well-being, and the conservation of biodiversity and ecosystems.

To reduce poverty and better use the city's natural resources, experts have studied factors affecting the performance of urban agriculture and found that culture, tradition, markets, availability of water, rainfall, and other elements typically influence the growth of urban agriculture. Other significant factors such as the soil type, plot size, land availability, proximity to the home, and access to finance are also determinant factors of urban agriculture. To overcome the problems, government policies and regulations can fund urban agriculture initiatives like rooftop gardens or community farming initiatives, which can advance social and environmental sustainability while also yielding a profit [38].

However, government policies and regulations are not the only factors contributing to urban agriculture's growth. Other factors that can influence the availability of land and water resources, the level of community support for urban agriculture, and the availability of technical expertise and training are all possible. Governments and stakeholders must address these other factors to promote urban agriculture. Working with community organizations and local businesses to identify and secure land and water resources for urban agriculture, providing training and technical assistance to urban farmers, and engaging the community to build support for urban agriculture initiatives are all examples of what this entails. In summary, sustainable finance can play a critical role in supporting the development of urban agriculture among micro and small enterprises by providing access to funding, technical assistance, and other resources that can promote sustainable food production and distribution in urban areas. Sustainable finance can also contribute to achieving broader sustainability goals and encourage the creation of more resilient, equitable, and sustainable food systems. Hence, the following H2 of the study assumes a positive relationship between urban agriculture and sustainable finance.

H2 Sustainable finance has a positive and significant effect on urban agriculture

H3 Sustainable finance has a significant mediating role between urban agriculture and government policies and regulations

**Fig. 1:**
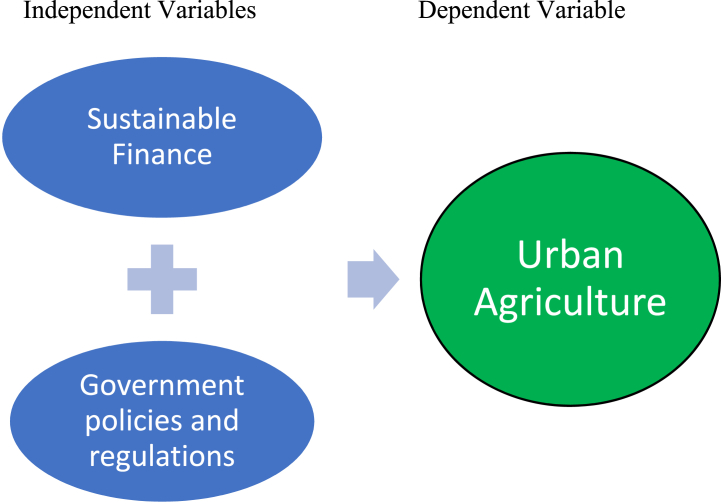
Conceptual framework of the study.

The following Fig. 1 of the study presents the conceptual framework, detailing both the dependent and independent variables. This framework provides a visual representation of the relationships and interactions among the key variables, offering a clear overview of the study's structure.

Independent Variables Dependent Variable.

## 3 Methodology and materials used

In this study, the role of government policies and regulations in promoting urban agriculture through sustainable finance was examined using explanatory and descriptive research designs with the help of a mixed research approach. The study's target population includes all operating micro and small businesses in Addis Ababa city administration. By 2021, the number of registered micro and small businesses in the city was 7667. The rationale behind choosing Addis Ababa city administration as a case study was that Addis Ababa is both the capital and the most developed urban area in Ethiopia. To determine the study's sample size, simple random sampling methods based on a margin of error of 5 % and a confidence interval of 95 % were used to calculate the study's sample size. As a result, 380 micro and small businesses are thus selected from the study's entire population. The study used the following Slovin's formula recommended by Ref. [39](Tejada et al., 2012), to determine the study's sample size.

$$n=N/\left(1+Ne^{2}\right)$$

N- Total population, e− Margin of error (5 %), n- Sample size.

Furthermore, the study used primary data to conduct an analysis. The primary data was used through questionnaires with close-ended questions with a Likert scale ranging from 1 to 5 (strongly agree to disagree strongly). The questionnaire was reverse coded and was prepared to measure the dependent variable (urban agriculture) with seven proxy measure questions such as land availability for urban agriculture, the current practice of regulations and policies, availability of water access to urban agriculture, availability of financial resources, availability of infrastructure, community support for urban agriculture, and finally the availability of Seeds and Supplies.

The target variable government policies and regulations are measured with five proxy questions identified from the reviewed literature. Those questions are land availability and zoning, water accessibility, financing, technical assistance, and market access. The other construct variable used as a mediating variable is sustainable finance. This variable is measured by eight proxy variables: affordability, accessibility, transparency, sustainability, inclusiveness, flexibility, market access, and environmental Stewardship. Different tools, including descriptive statements, frequency distribution, and SMART-PLS software, were used to analyze and discuss the data gathered through the questionnaire distributed to respondents. The following equation (1) of the study illustrates the estimated model used to examine the relationship between the construct variables.

(1) U.A.=f(GPR,S.F)

Whereas, U.A. ∼ urban agriculture (measured by proxy questions such as urban agriculture land availability, regulations and policies, urban water access, financial resources, infrastructure, and community support with a Likert scale approach)

GPR ∼ government policies and regulations (measured by proxy questions such as policies and regulations on urban agriculture land availability and zoning (LAZ), water accessibility (W.A.), financing (F.I.), Technical Assistance (T.A.), and market access (M.A.) with a Likert scale approach)

S.F. ∼sustainable finance (measured by proxy questions such as sustainable finance affordability (SFA), accessibility (SFAC), transparency (SFT), sustainability (SFS), inclusiveness (SFI), flexibility (SFF), market access (SFMA), and environmental and Stewardship (SFES) with a Likert scale approach).

Given this context, Equation (2) presents the econometric model employed to analyze the relationship between the variables under investigation. This model is crucial for understanding the dynamics and interactions within the data, providing a statistical framework to test hypotheses and interpret results accurately.

(2) Modelequation=UAt=β0+β1LAZt+β2WAt+β3FIt+β4TAt+β5MAt+β6SFAt+β7SFACt+β8SFTt+β9SFSt+β10SFIt+β11SFIt+β12SFFt+β13SFMAt+β14SFESt+c

Note: β1 – β5 ∼ Government policies and regulations proxy questions, β6 – β14 ∼ Sustainable finance proxy questions.

## 4 Results and discussion

Three hundred eighty questionnaires were given out as part of the study to chosen respondents from micro and small businesses, and 324 of them were completed and returned (85.3 percent response rate). The study's descriptive statistics will be discussed in the following section, including descriptive statements. The following (Table 1) survey shows the descriptive statistics of each construct variable used in the study.

**Table 1:** Descriptive statistics of Government policies and regulations.

Government policies and regulations	
Land Availability and Zoning (LAZ)	Government Policies and regulations allow for the allocation of urban land for agriculture and provide clear guidelines for using urban agricultural land.
Water Accessibility (W.A.)	Access to water for urban agriculture is secured through policies and regulations that ensure equitable distribution and use of resources.
Financing (F.I.)	The government provides enough financing options for micro and small businesses engaged in urban agriculture, including low-interest loans and tax incentives.
Technical Assistance (T.A.)	The government provides technical assistance and support to help micro and small businesses engaged in urban agriculture integrate sustainable practices into their operations.
Market Access (M.A.)	Government policies facilitate market access for micro and small businesses engaged in urban agriculture by promoting direct sales to consumers, institutions, and other companies.

	Minimum	Maximum	Mean	Std. Deviation
**Government Policies and Regulations**				
Land Availability and Zoning	2.00	5.00	3.6400	0.83245
Water Accessibility	2.00	5.00	3.7600	0.80270
Financing	1.00	5.00	3.4533	0.96273
Technical Assistance	2.00	5.00	3.6400	0.89503
Market Access	1.00	5.00	3.2800	0.95238
Valid N (listwise)	324			

All proxy questions used to measure the latent variables of the study are operationalized as an indirect Likert approach from strongly disagree (5) to agree (1). This approach provides a better understanding and clarity of the study questions. Hence, the study used direct implications to present and discuss the study's findings. Consequently, the study's next section covers the descriptive statistics of each indicator. To evaluate the overall response value of each hand used under each variable, the guideline offered by Ref. [40] was applied in this study. The author claims that the mean value of the indicators can be used to understand the explanatory power of each hand. Indications with a mean score of 1–2.61 are classified as having the lowest mean score, 2.62–3.41 as average or moderate, 3.42–4.21 as excellent or high, and 4.22–5 as very good.

The study's target variable is government policies and regulations relating to urban agriculture. Five proxies are used to quantify this variable to examine how these policies and regulations support the growth of urban agriculture. The first proxy question focuses on (Land Availability and Zoning) and demonstrates how government policies permit the distribution of urban land for agriculture and set forth precise criteria for the usage of urban agricultural land. This question has a mean score of 3.64 and a standard deviation of 0.83. According to Ref. [40], the overall average Land Availability and Zoning score are high under these criteria. However, this variable is operationalized as an indirect implication to bring a more meaningful and clear understanding of the lack of supportive government rules and regulations regarding allowing agricultural land and zoning them. Hence, the overall respondent's response confirms that there is a lack of government Policies that allow for the allocation of urban land for agriculture and provide clear guidelines for using urban agricultural land.

The second proxy question focused on (Water Accessibility), which examines whether policies and regulations guarantee equitable resource distribution and use have been implemented to assure access to water for urban agriculture. Among the various questions offered for this variable, this question item had the most excellent mean value with a mean score of 3.7. The government's ability to finance micro and small companies engaged in urban agriculture through low-interest loans and tax advantages was the subject of the third proxy question of government policies and regulations (Financing). The average score for this response was 3.4. Technical assistance was used as a fourth proxy question to see if the government provides technical assistance and support to help micro and small businesses engaged in urban agriculture integrate sustainable practices into their operations. This item scored a mean value of 3.64. Finally, market Access was used as the fifth proxy measure of government policies and regulations regarding urban agriculture. This item was used to investigate if government policies facilitate market access for micro and small businesses engaged in urban agriculture by promoting direct sales to consumers, institutions, and other companies. This item scored a mean value of 3.2.

Generally, based on guidelines offered by Ref. [40], all questions used to measure government policies and regulations scored the highest mean value, which means the respondents disagree with the questions provided under this variable. Based on the response rate, the issues of water accessibility, land availability, zoning, Technical Assistance, financial issues, and market access are the main problems of urban agriculture that shall be solved through government policies and regulations. The following Table 2 of the study shows the descriptive statistics of each observed variable under the latent variable of sustainable finance.

**Table 2:** Descriptive statistics of Sustainable Finance.

Sustainable Finance	
Affordability (SFA)	The financing options are accessible and affordable for micro and small businesses engaged in urban agriculture, with reasonable interest rates and repayment terms.
Accessibility (SFAC)	The financing options are easily accessible to many micro and small businesses engaged in urban agriculture, regardless of size, location, or sector.
Transparency (SFT)	The financing process is transparent and clearly understood by the borrowers, including the terms and conditions, costs, and requirements.
Sustainability (SFS)	The financing supports adopting sustainable practices in urban agriculture and contributes to achieving environmental and social sustainability goals.
Inclusiveness (SFI)	The financing options are available to various micro and small businesses engaged in urban agriculture, including those owned by women, youth, and marginalized communities.
Flexibility (SFF)	The financing options are flexible, adapting to the specific needs and circumstances of micro and small businesses engaged in urban agriculture and allowing for changes over time.
Market Access (SFMA)	The financing options support the development of marketing and distribution channels for micro and small businesses engaged in urban agriculture to increase their access to markets.
Environmental Stewardship (SFES)	The financing options encourage and support adopting environmentally sustainable practices in urban agriculture, such as using organic methods and conserving natural resources.

Sustainable Finance	Minimum	Maximum	Mean	Std. Deviation
Affordability	2.00	5.00	3.3467	0.89281
Accessibility	1.00	5.00	3.6400	0.83245
Transparency	1.00	5.00	3.3467	0.87755
Sustainability	1.00	5.00	3.4933	0.82811
Inclusiveness	2.00	5.00	3.6667	0.81096
Flexibility	2.00	5.00	3.7200	0.90882
Market Access	2.00	5.00	3.9067	0.73839
Environmental Stewardship	2.00	5.00	4.1867	0.65126
Valid N (listwise)	324			

This study employed sustainable finance as the mediating variable to examine how governmental policies and regulations impact the promotion of urban agriculture. To determine the mediating role, sustainable finance was concerned using proxy questions. The first proxy question relates to (Affordability), which demonstrates how financing options are available for micro and small firms involved in urban agriculture, with fair interest rates and repayment terms. This question scored an overall mean value of 3.34. The overall average score of sustainable finance affordability is high. Hence, the respondent's response on the lack of sustainable finance affordability is argued.

The second proxy question focused on the (Accessibility) of sustainable finance, which indicates how various micro and small firms participating in urban agriculture may easily access financial access regardless of size, location, or industry (3.64 mean value). The third question focused on the (Transparency) of sustainable finance, demonstrating how the borrowing process is open to the borrowers and that they understand the terms and conditions, fees, and obligations (3.34 mean value). The fourth proxy question focuses on the (Sustainability) of finance and asks how financing helps urban agriculture adopt sustainable practices and advances environmental and social sustainability objectives (3.5). Inclusiveness is also used as the fifth proxy question to measure sustainable finance. Inclusiveness addresses financing options available to various micro and small businesses engaged in urban agriculture, including those owned by women, youth, and marginalized communities (3.67). The sixth proxy question of sustainable finance is related to flexibility), which shows how the financing options are flexible, adapting to the specific needs and circumstances of micro and small businesses engaged in urban agriculture and allowing for changes over time (3.72).

Market Access is the seventh proxy question that addresses how the financing options support the development of marketing and distribution channels for micro and small businesses engaged in urban agriculture to increase their access to markets (3.92). The last proxy question is related to environmental Stewardship), which is used to answer how the financing options encourage and support adopting environmentally sustainable practices in urban agriculture, such as using organic methods and conserving natural resources (4.2). The following Table 3 of the study shows the descriptive statistics of each observed variable under the latent variable of Urban Agriculture.

**Table 3:** Descriptive statistics of urban agriculture.

Urban Agriculture	
Land Availability	Access to suitable and adequate land in urban areas is crucial for developing urban agriculture.
Regulations and Policies	Government policies and regulations can significantly impact the development of urban agriculture, both positively and negatively.
Urban Water Access	Access to water, both for irrigation and processing, is essential for the development of urban agriculture.
Financial Resources	The lack of financial resources can limit the development of urban agriculture, including the availability of loans and grants for urban farmers.
Infrastructure	Various factors, such as transportation costs and competition from large-scale agriculture, limit market access to sell urban agriculture products.
Community Support	The support and involvement of the urban community are crucial for developing urban agriculture, including participation in decision-making and providing volunteer labor and resources.
Availability of Seeds and Supplies	The availability of seeds, fertilizer, and other supplies is necessary to develop urban agriculture.

Urban Agriculture				
	Minimum	Maximum	Mean	Std. Deviation
Land Availability	2.00	5.00	4.4133	0.61717
Regulations and Policies	2.00	5.00	3.6400	0.83245
Urban Water Access	2.00	5.00	3.7600	0.80270
Financial Resources	1.00	5.00	3.4533	0.96273
Infrastructure	2.00	5.00	3.6400	0.89503
Community Support	1.00	5.00	3.2800	0.95238
Availability of Seeds and Supplies	2.00	5.00	3.3467	0.89281
Valid N (listwise)	324			

The mean value for the overall response rate for each indicator related to the urban agriculture variable is more than 3.42 except for two proxy questions (community support, which is used to investigate if there is the support and involvement of the urban community for the development of urban agriculture, including participation in decision-making and the provision of volunteer labor and resources. Moreover, the Availability of Seeds and supplies is used to investigate the availability of seeds, fertilizer, and other supplies necessary for the development of urban agriculture). The mean value of this item is 3.28 and 3.33, respectively, close to 3 (scaled as neutral). This result is an implication for community support, and the Availability of Seeds and Supplies is not found to be an issue for micro and small enterprises involved in urban agriculture.

The other indicators under this variable have a mean value of greater than 3.42, and each hand in this example has a mean value near 4, which is classed as disagreeing with the questions related to urban agriculture, which is supposed to suggest that the indicators are in disagreement. Therefore, it can be understood that the issue of (Land Availability), is used to access suitable and adequate land in urban areas to develop urban agriculture. (Regulations and Policies) which is used to investigate Government policies and regulations that can significantly impact the development of urban agriculture, both positively and negatively. (Water Access) which is used to access water, both for irrigation and processing, is essential for the development of urban agriculture. (Financial Resources) which is used to investigate the need for more financial resources that can limit the growth of urban agriculture, including the availability of loans and grants for urban farmers. (Infrastructure) which is used to access markets for the sale of urban agriculture products, is limited by various factors, such as transportation costs and competition from large-scale agriculture.

### 4.1 Measurement model of the study

To look at the connections between the latent variables and their measures, the study used an outer (measurement) model. Before analysis, items assessing sustainable finance, government policies and regulations, and urban agriculture were reverse-coded to make them positive so that, during path modeling, all things from all components would be moving in the same direction (positive). Since each measure has factor loading values greater than 0.70, it is determined that the outer loading of the standards is substantially connected with the construct variables [41]. Additionally, composite reliability and Cronbach alpha were used to carry out the reliability analysis that is supposed to be the initial part of the measurement model. The cutoff threshold for Cronbach alpha and composite reliability is expected to be higher than 0.70, following the benchmark set by Ref. [42]. Therefore, the reliability analysis test result indicates that all variables have a test result more significant than the cutoff point (Table 4). The second criterion of the measurement model is related to convergent validity; convergent validity helps determine data validity with an average variance extracted value (AVE). According to average variance criteria, each latent variable should score greater than 0.50 AVE to fulfill convergent validity [42]. Based on this fact, the study concluded that the data has no issue with convergent validity as the model has a more significant 0.50 cutoff point (Table 4). The case of collinearity was tested through the variance inflation factor. According to Ref. [42], the variance inflation factor should be less than 10 to ignore the collinearity issue. Hence, based on this argument, the study concluded that there is no evidence of collinearity between variables since the value of the variance inflation factor is less than 10 for all variables (Table 4). Table 4 of the study shows the test results of Reliability analysis, convergent validity, and collinearity statistics.

**Table 4:** Reliability analysis, convergent validity, and collinearity statistics.

	Outer L	VIF	CR	AVE	C.Alpha
**Government Policies and Regulations**					
Land Availability and Zoning	0.852	1.973	0.896	0.633	0.856
Water Accessibility	0.856	2.648			
Financing	0.737	1.887			
Technical Assistance	0.784	2.235			
Market Access	0.771	2.841			
**Sustainable Finance**					
Affordability	0.734	1.962	0.917	0.581	0.896
Accessibility	0.717	5.00			
Transparency	0.802	2.440			
Sustainability	0.823	2.199			
Inclusiveness	0.704	2.400			
Flexibility	0.745	2.003			
Market Access	0.805	1.853			
Environmental Stewardship	0.757	2.587			
**Urban Agriculture**					
Land Availability	0.825	2.160	0.910	0.590	0.884
Regulations and Policies	0.745	2.401			
Urban Water Access	0.772	2.210			
Financial Resources	0.712	1.605			
Infrastructure	0.768	2.391			
Community Support	0.780	2.150			
Availability of Seeds and Supplies	0.742	1.898			
Valid N (listwise)	324				

The other criterion used to test the measurement model is discriminant validity. Discriminant validity can be tested with a different approach; however, the most known method is the heterotrait-monotrait ratio. According to the heterotrait-monotrait ratio, the latent variable of the study should have a cut point of less than 0.80 percent to validate the discriminant validity. Based on this fact, this study has no evidence of reporting that the data violates the discriminant validity criteria (Table 5). Table 5 of the study shows the test result of discriminant validity.

**Table 5:** Reporting discriminant validity through Heterotriat-monotrait ratio.

Latent Variable	GPR	SF	U.A.
Government Policies and Regulations			
Sustainable Finance	0.367	0.438	
Urban Agriculture	0.438		

### 4.2 Structural model analysis

The study investigated the linkage between the proposed variables using structural model assessment. In doing so, the authors examined both the direct, which explores how sustainable finance and government policies affect urban agriculture, and the indirect effect to investigate how government policies and regulations affected the promotion of urban agriculture using sustainable financing as a mediating variable. As specified in the study's hypothesis. H1 of the study examines whether government policies and regulations positively and significantly impact urban agriculture. The outcome of the structural model analysis suggests that government policies and regulations have an insignificant impact on the progress of urban agriculture. The path coefficient (β=0.059,t−statistics=1.165p−value=0.245) suggests that existing government policies and regulations do not favor promoting urban agriculture in Ethiopia. However, this variable significantly and positively influences the amount of sustainable finance. There could be several possible reasons why existing government policies and regulations do not favor promoting urban agriculture in Ethiopia. Among many others, Lack of awareness or understanding, Prioritization of other sectors, Limited capacity and expertise, Economic considerations, Land tenure issues, Resistance from other stakeholders, Lack of coordination and collaboration, and Political factors are considered as a main factor.

On the other hand, the study's path coefficient (β=0.858,t−statistics=35.217,p−value=0.000) suggests that government policies and regulations are more important in supporting sustainable financing than urban agriculture. The working hypothesis does not support the findings of the study. There are several possible reasons why government policies and regulations are considered more important in supporting sustainable financing than urban agriculture. Firstly, the government may prioritize economic development and revenue generation, seeing sustainable financing as a means to support overall economic growth [43]. Urban agriculture may be viewed as a smaller contributor to the economy compared to other sectors that generate more revenue. Additionally, limited resources could play a role, with the government having limited funding available for supporting initiatives [44]. Therefore, they may prioritize policies and regulations that have a broader impact on the economy rather than focusing specifically on urban agriculture. Moreover, the government may perceive urban agriculture as a risky investment due to uncertainties related to market demand, climate change, and potential conflicts with existing land-use plans. They may view sustainable financing as a more stable and predictable avenue for attracting investments. Furthermore, international obligations could influence prioritization, with the government focusing on meeting commitments related to sustainable financing outlined in international agreements [45]. Political considerations may also come into play, with pressure from influential stakeholders or interest groups influencing policy decisions. Additionally, a lack of awareness or understanding could lead to a focus on other sectors that are perceived as having more immediate economic benefits. Institutional capacity and public perception could also contribute to the prioritization of sustainable financing over urban agriculture [46].

As it can be seen from Table 6 of the study, hypothesis testing and model summary, H2 of the study assesses whether the promotion of urban agriculture is positively and significantly impacted by sustainable finance. According to the structural model analysis, urban agriculture is positively and significantly affected by sustainable finance, with a path coefficient of (β=1.032,t−statistics=23.853,andp−value=0.000). The study's finding suggests that providing sustainable finance for micro and small enterprises engaged in urban agriculture will significantly improve urban agriculture's performance. The mediating role of sustainable finance between urban agriculture and government policies and regulations is investigated through H3 of the study. As a result, the structural model analysis with path coefficient (β=0.885,t−statistics18.443,pvalue0.000) shows a positive and significant mediating role for sustainable finan ce. It means that government policies and regulations impact urban agriculture through sustainable financing. The result of structural model estimation suggests an indirect effect of government policies and regulations on urban agriculture. This result implies that all government policies and regulations shall focus on enhancing sustainable finance for micro and small businesses to promote urban agriculture. Sustainable finance fully mediates between government policies and regulations and urban agriculture. The positive and significant mediating role of sustainable finance between urban agriculture and government policies and regulations can be attributed to several factors. Firstly, the increased availability of sustainable finance options such as green bonds, impact investing, or grants has made funding more accessible for urban agriculture initiatives [46]. This influx of financial resources allows these projects to thrive, thereby influencing government policymakers to recognize their potential and enact favorable policies and regulations. Furthermore, the presence of sustainable finance in urban agriculture projects enhances their credibility and legitimacy [45]. When government policymakers witness successful initiatives backed by sustainable finance, they are more likely to support and implement supportive policies and regulations. This credibility factor plays a crucial role in influencing government decisions. Moreover, sustainable finance contributes to the economic viability of urban agriculture projects. By providing financial resources, it enables the development of efficient and sustainable agricultural practices, leading to increased productivity, job creation, and economic growth [47]. Recognizing these economic benefits, governments are motivated to enact policies and regulations that promote the expansion of urban agriculture. Additionally, sustainable finance supports the environmental and social impact of urban agriculture [43]. With its focus on sustainable practices, urban agriculture can have positive outcomes such as eco-friendly technologies, waste reduction, and community engagement. By providing resources for these practices, sustainable finance showcases the positive outcomes of urban agriculture projects to governments, leading them to implement policies and regulations that encourage their growth. In summary, the presence of sustainable finance in urban agriculture projects creates a positive feedback loop. Increased availability of funding options, enhanced credibility and legitimacy, economic benefits, and positive environmental and social impacts all contribute to improved government policies and regulations. This, in turn, further promotes sustainable finance in the sector, creating a mutually beneficial relationship between urban agriculture and government support. Hence, more consideration must be given to enhancing sustainable finance to promote urban agriculture performance. Table 8 of the study shows the indirect estimation of the structural model.

**Table 6:** Hypothesis testing and model summary.

Hypothesis	Direct effect (Beta)	SD	T-statistics	P-value
H1: GPR→→UA	−0.059	0.051	1.165	0.245
GPR→→SF	0.858	0.024	35.217^a^	0.000
H2: SF→→UA	1.032	0.043	23.853^a^	0.000

**Note:***t > 3.29 at*.

a *p* < *0.001*.

**Table 7:** Result of moderating analysis.

Hypothesis	Indirect effect	SD	T-statistics	P-value
H3: GPR→SF→UA	0.885	0.048	18.443^a^	0.000

**Note:***t > 3.29 at*.

a p < 0.001.

**Table 8:** The result of the model fit.

Variables	R-square	Adjusted R-square	SRMR
Sustainable Finance	0.736	0.732	0.0136
Urban Agriculture	0.964	0.963	

Note: SRMR- Standardized Root Mean Square Residual.

The study also examines the role of government policies and regulations in promoting urban agriculture through sustainable finance, as evidenced by micro and small enterprises in Ethiopia. The above Table 7 of the study shows the result of moderating analysis. In this regard, most of the previously conducted studies are more or less stacked to examine urban agriculture determinants at the macro and micro levels. However, seeing how government policies and regulations in the framework of sustainable finance affect urban agriculture is essential, especially for developing economies where there needs to be more encouragement for urban agriculture. The study found a positive and significant indirect effect of government regulations and policies on urban agriculture with a coefficient (β=0.885,t−statistics=18.443,p−value=0.000). Hence, H3 of the study is accepted. However, in the direct structural model estimation, government policies and regulations have a negative and insignificant effect on promoting urban agriculture among micro and small enterprises found in Ethiopia. This result indicates that the current practice of policies and regulations related to urban agriculture is not enough to promote sustainable urban agriculture.

The negative relationship results as specific zoning laws and regulations restrict urban land use for agriculture and make it difficult for urban micro and small businesses to find suitable land for growing crops. The government's lack of funding and financial incentives is also a barrier to urban agriculture, as responded by respondents. Furthermore, the issue of access to water is limited in urban areas, and regulations around water usage restrict the amount of water that urban farmers can use for irrigation. Overall, government policies and regulations in Ethiopia create obstacles and challenges for urban agriculture, making it more difficult for urban micro and small enterprises and farmers to grow crops and provide fresh food to their communities.

To that end, sustainable finance and urban agriculture have a positive and significant relationship with a P-value of 0.000. The study's findings confirm the study's hypothesis claim that sustainable finance has a positive and significant impact on urban agriculture. The positive link suggests that urban agriculture can be encouraged if sufficient sustainable financing is given to micro and small firms involved in urban agriculture. As a result, sustainable funding may support local food systems, boost the accessibility of fresh, homegrown food in urban areas, and provide cash and capital to urban agriculture. Enhancing sustainable finance also supports the development of urban agriculture by helping to create jobs and spur economic growth in urban areas.

Furthermore, urban agriculture can benefit from greater support from sustainable finance, enabling farmers to spend money on infrastructure, tools, and other resources to grow crops and make food in an eco-friendly way. Sustainable financing can also make it easier for farmers to establish and expand their businesses by increasing access to credit for urban agriculture. Environmentally friendly behaviors, including using renewable energy, cutting back on trash, and conserving water, are encouraged by sustainable finance. This can support sustainability and lessen the damaging effects of agriculture on the environment. Hence, the study accepts H2, and H3 with *coefficient* (β=0.885,t−statistics=18.443,p−value=0.000),and(β=1.032,t−statistics=23.853,p−value=0.000) respectively.

### 4.3 Model fit

**Fig. 2:**
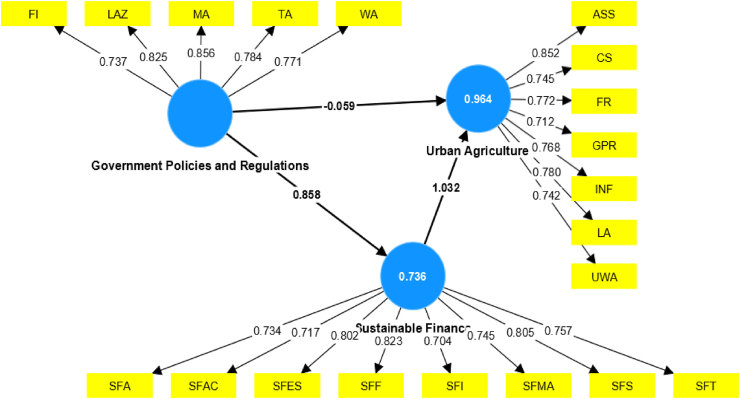
Output of Model summary.

Model-appropriate measures how well a statistical model captures the underlying patterns in the data. A good model fit captures the key trends and relationships in the data, and the model's predictions are close to the actual values [48]. The amount of variance in the dependent variable explained by the independent variables in a regression model is measured statistically as R-squared. The range of R-squared is 0–1, with a value of 1 denoting that the model perfectly accounts for all the variability in the dependent variable and a value of 0 denoting that the model accounts for none of the variability in the dependent variable. The R square was used to determine how much of the dependent variable is explained by the independent variables in this study [49], which measures the model's goodness of fit [50]. As a result, sustainable finance and government policies and regulations explained the dependent variable of the study of urban agriculture by 96.4 percent. Other variables will present the remaining 3.6 percent. The following Fig. 2 of the study displays the overall output of the model fit and the outer loadings of each variable. This figure provides a comprehensive overview of how well the model fits the data and the strength of the relationships between the observed variables and their corresponding latent constructs.

The other criterion used to measure the model fit was Standardized Root Mean Square Residual (SRMR), a measure of model fit in structural equation modeling (SEM). It estimates the difference between the observed covariance matrix and the covariance matrix implied by the model. A good model fit for SRMR is indicated by a value close to 0. A value below 0.08 is generally acceptable, while values above 0.10 suggest that the model may not fit the data well. As a result, the study is fine with model fit with both R-square and Standardized Root Mean Square Residual measures fulfilling the criteria. Furthermore, the following bar graph shows that the R-squared value is usually represented as a horizontal bar, where the bar's length indicates the R-squared value's magnitude. The higher the R-squared value, the better the model fits the data, and vice versa.

**Fig. 3:**
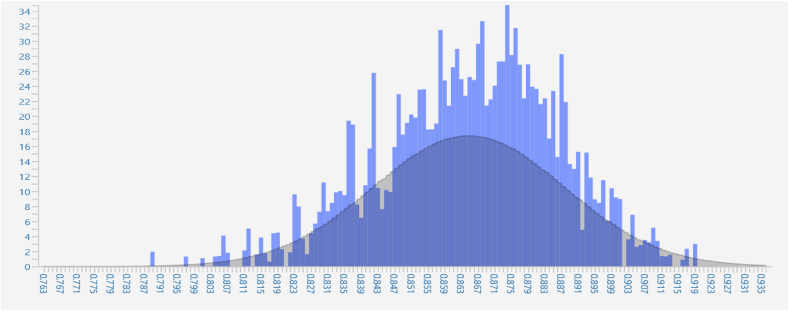
The result of the normality test (Histogram).

In addition, the histogram can be used to measure the model's fitness. Under these criteria, the residuals should be normally distributed if the model is a good fit, with a mean of zero and a constant variance. It can be seen in the histogram as a bell-shaped curve centered around zero. Suppose the residuals are not normally distributed, or there are outliers or other anomalies. In that case, it may indicate that the model is not a good fit for the data. The following Fig. 3 of the study presents the results of the normality test. This figure illustrates whether the data distribution meets the assumptions of normality, which is crucial for the validity of many statistical analyses conducted within the study. As a result, there is no issue with the normality test as the histogram is bell-shaped.

## 5 Conclusion

One of the most crucial challenges in emerging nations is promoting urban agriculture [51]. Enhancing urban agriculture has been shown to increase food security in cities. It is strongly asserted that this industry needs to attract more individual farmers, private investors, and micro and small firms [52]. Government policies and regulations can promote urban agriculture by providing incentives, such as tax breaks or subsidies, to encourage investment in urban agriculture initiatives. Additionally, they can set standards for environmentally sustainable practices and provide funding for research and development of new technologies supporting urban agriculture. Regulations can also help ensure access to land, water, and other resources and food safety. By providing support and resources, governments can help to make urban agriculture a viable and sustainable means of food production [45]. In doing so, providing sustainable financing to support urban agriculture is crucial in this process. As a result, sustainable finance may assist farmers in raising their production and also give them the money they need to invest in environmentally friendly farming techniques like precision farming, organic farming, and conservation tillage. These techniques can boost crop yields and reduce inputs like water and fertilizer, making farming operations more effective and fruitful [51].

This study investigates the effect of government policies and regulations on urban agriculture, using sustainable finance as a mediating variable. The finding of the study implies that the current policies and rules practiced in Ethiopia are not found to be an urban agriculture promoter. The two latent variables (urban agriculture and government policies and regulations) have a negative and insignificant relationship. However, the mediating variable of sustainable finance significantly contributes to promoting urban agriculture. At the same time, the study found the full mediating role of sustainable finance between urban agriculture and government policies and regulations.

As a way to conclude, the relationship between sustainable finance and urban agriculture is positive, with sustainable finance having the potential to impact and support the growth of urban agriculture significantly. Sustainable finance can advance urban agriculture and create a more sustainable future by providing increased funding, increasing access to capital, promoting environmentally friendly practices, stimulating economic growth, and supporting local food systems. However, it is essential to note that sustainable finance is just one aspect of a more considerable effort to promote and support urban agriculture. Additional measures, such as education and outreach programs, policy support, and community engagement, are necessary to create a supportive environment for urban agriculture to thrive. The fact that this study only included micro and small business owners in Addis Ababa city administration as it is challenging to conduct the study at the country level. Hence, future research could extend this investigation to regional areas of the country. Based on the study's findings, it is advised that all concerned parties support urban agriculture by enacting laws and regulations that encourage the development of micro and small businesses engaged in urban agriculture. When developing policies and regulations that deliberately affect urban agriculture, the government considers packages such as tax breaks, subsidies, and land use laws. It is also recommended to promote innovation and entrepreneurship through sustainable finance. Universities, research facilities, and other organizations can contribute to developing new technology and best practices for urban agriculture while encouraging partnerships between micro and small companies.

## Funding

The APC was funded by the Hungarian University of Agriculture and Life Science, Doctoral School of Economic and Regional Sciences.

## Data availability statement

The data can be available from request goshudasalegn@gmail.com.

## Use of AI tools declaration

The authors declare they have not used Artificial Intelligence (AI) tools in the creation of this article.

## CRediT authorship contribution statement

**Goshu Desalegn:** Writing – review & editing, Writing – original draft, Software, Resources, Methodology, Formal analysis, Data curation, Conceptualization. **Anita Tangl:** Visualization, Validation, Supervision, Project administration, Investigation, Data curation. **Maria Fekete-Farkas:** Supervision, Resources, Project administration, Investigation, Funding acquisition, Conceptualization. **Girma Gudisa:** Writing – review & editing, Writing – original draft, Formal analysis, Data curation, Conceptualization. **Anita Boros:** Visualization, Supervision, Resources, Project administration, Investigation, Funding acquisition, Formal analysis.

## Declaration of competing interest

The authors declare the following financial interests/personal relationships which may be considered as potential competing interests: Goshu Desalegn reports article publishing charges was provided by Hungarian University of Agriculture and Life Sciences. If there are other authors, they declare that they have no known competing financial interests or personal relationships that could have appeared to influence the work reported in this paper.
